# Supplementary material for: A novel environment-evoked transcriptional signature predicts reactivity in single dentate granule neurons
Source: Nat Commun. 2018 Aug 6;9:3084. doi: 10.1038/s41467-018-05418-8 (PMC6079101; doi:10.1038/s41467-018-05418-8)
Supplement: Supplementary file 1 — Supplementary Information [file 41467_2018_5418_MOESM1_ESM.pdf]

Supplementary Information

A novel environment-evoked transcriptional signature predicts reactivity in single dentate granule neurons

Jaeger, Linker, Parylak, *et al*.

Supplementary Figure 1

Strategy to identify an endogenous transcriptional signature that primes neurons for reactivity

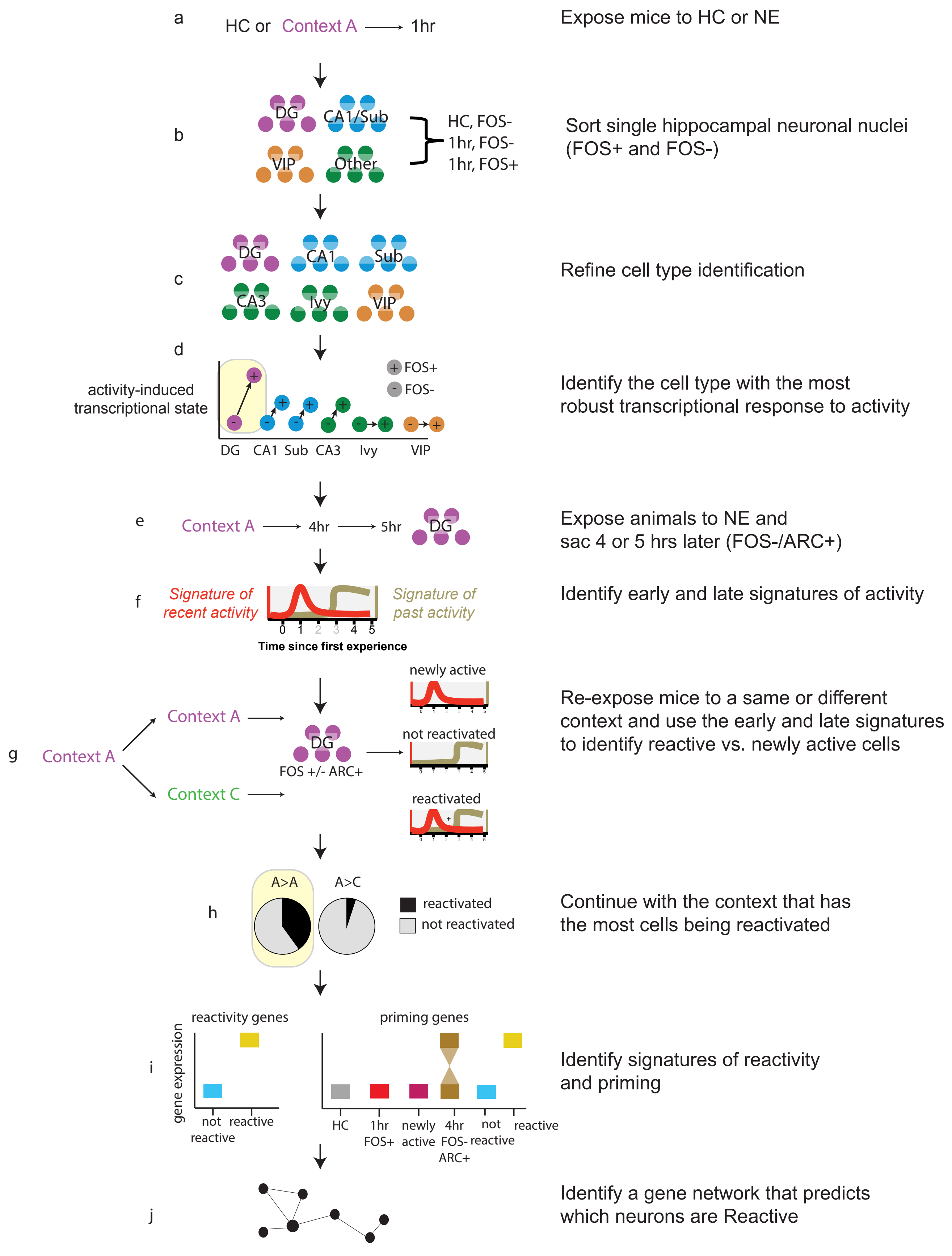

Supplementary Figure 1: ***Strategy to identify reactivity signature***

Overview of the study design to identify (1) the cell type with the largest transcriptional change in response to activity, (2) the long-term dynamics of activity-induced change, and (3) if a transcriptional signature is preferentially enriched in neurons that are reactivated upon a second exposure. a) Mice were first either placed in context A or remained in the HC. One hr later hippocampal neurons were dissociated. b) Neurons were then initially labeled with hippocampal neuronal markers NEUN, PROX1 and CTIP2 to discriminate major known cell types. c) SnRNA-seq then enabled a refinement of cell type identification. d) DEGs between FOS+ and FOS- nuclei were calculated within the refined cell populations, and the cell type with the largest response in FOS+ neurons (DG) was used for downstream analysis. e) Mice were then exposed to context A and returned to the HC for 4 or 5 hr. DG nuclei were sorted for ARC+FOS- nuclei to identify previously active neurons. f) Early and late gene signatures were then identified by comparing the signatures at 1, 4 or 5 hrs. g) Mice were then exposed to two contexts, either A>A or A>C. DG neurons were sorted on ARC and FOS protein and compared to the early and late signatures to identify Newly Activated and Reactivated neurons. h) The population with the largest proportion of Reactivated neurons (A>A) was used to determine the predictive signature. i) Genes were filtered for those that had a high likelihood of predicting reactivity, and a classification model was produced to perform predictions. j) Ultimately, a set of genes was identified that, when combined, were highly predictive of reactivity.

Supplementary Figure 2

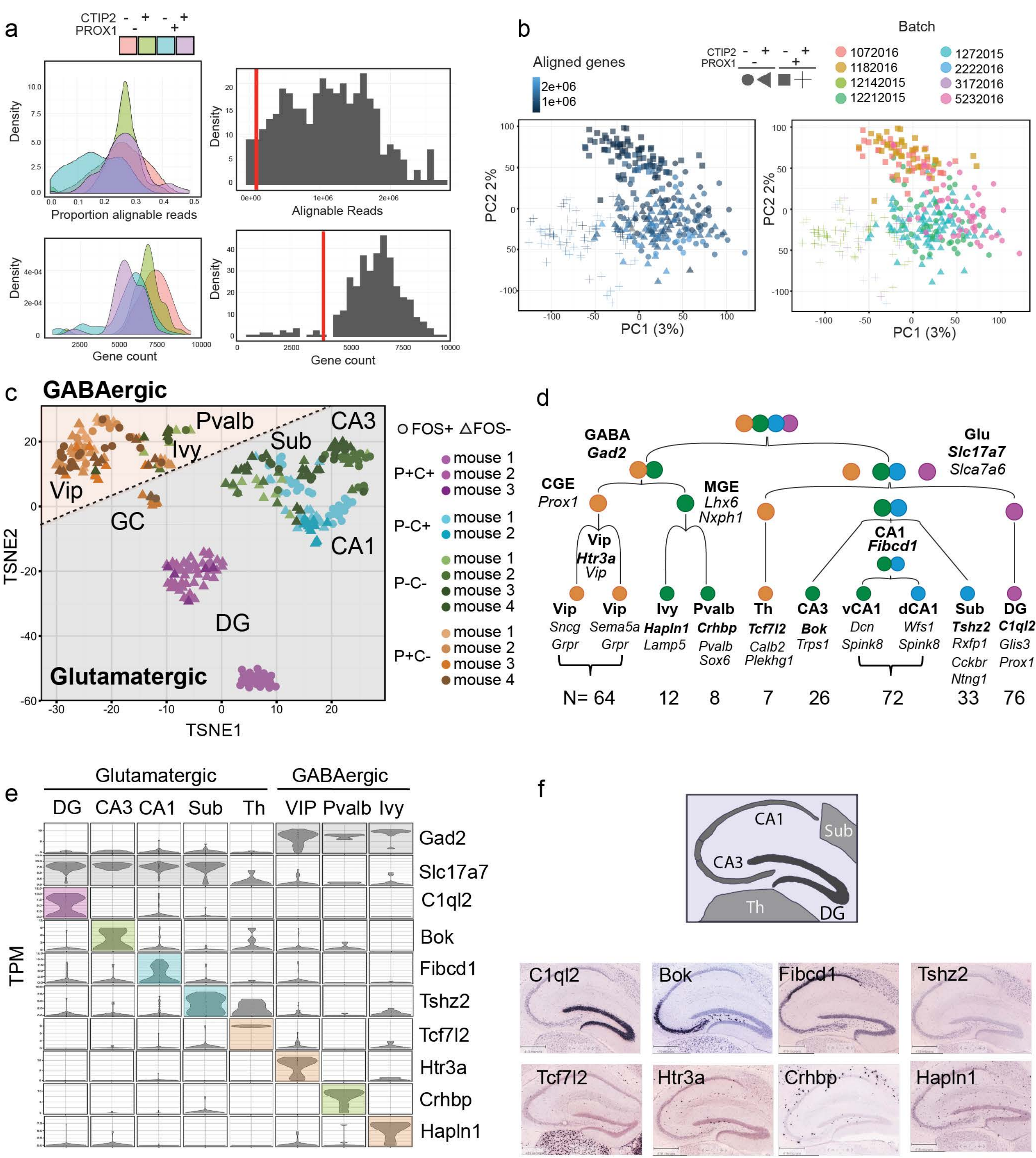

Supplementary Figure 2: ***Overview of quality control measures***

a) (Top left) Distribution of alignable reads per nucleus colored by protein marker combination. (Top right) Distribution of total alignable reads for all nuclei with the cutoff placed at 100,000 reads. (Bottom left) Distribution of gene counts per nucleus colored by protein marker combination. (Bottom right) Total gene count for all nuclei with cutoff placed at 4,000 genes. b) (Left) PCA of all samples colored by aligned reads and shaped by protein marker combination. (Right) PCA of all samples colored by batch date and shaped by protein stain. c) T-SNE plot of hippocampal nuclei from both HC and NE conditions. PROX1+CTIP2+ (P+C+; pink), PROX1-CTIP2+ (P-C+; blue), PROX1-CTIP2- (P-C-; green); and PROX1+CTIP2- (P+C-; orange) populations were each isolated from multiple mice that are represented by different saturations of color. d) Flow of group membership for all nuclei. The top level splits all nuclei into GABAergic and glutamatergic. The bottom nodes represent the predicted cell type. N = number of nuclei in each final group. CGE = caudal ganglionic eminence, MGE = medial ganglionic eminence, vCA1 ventral CA1, dCA1= dorsal CA1, Sub = Subiculum e) Representative violin plots for genes that are uniquely expressed within a given hippocampal cell type (Random forest padj < 0.05). f) Schematic of the mouse hippocampus with the subiculum (Sub) CA1, CA3, dentate gyrus (DG), and thalamus (Th) noted. *In situ* images from the Allen Brain Atlas show expression patterns of genes from panel e.

Supplementary Figure 3

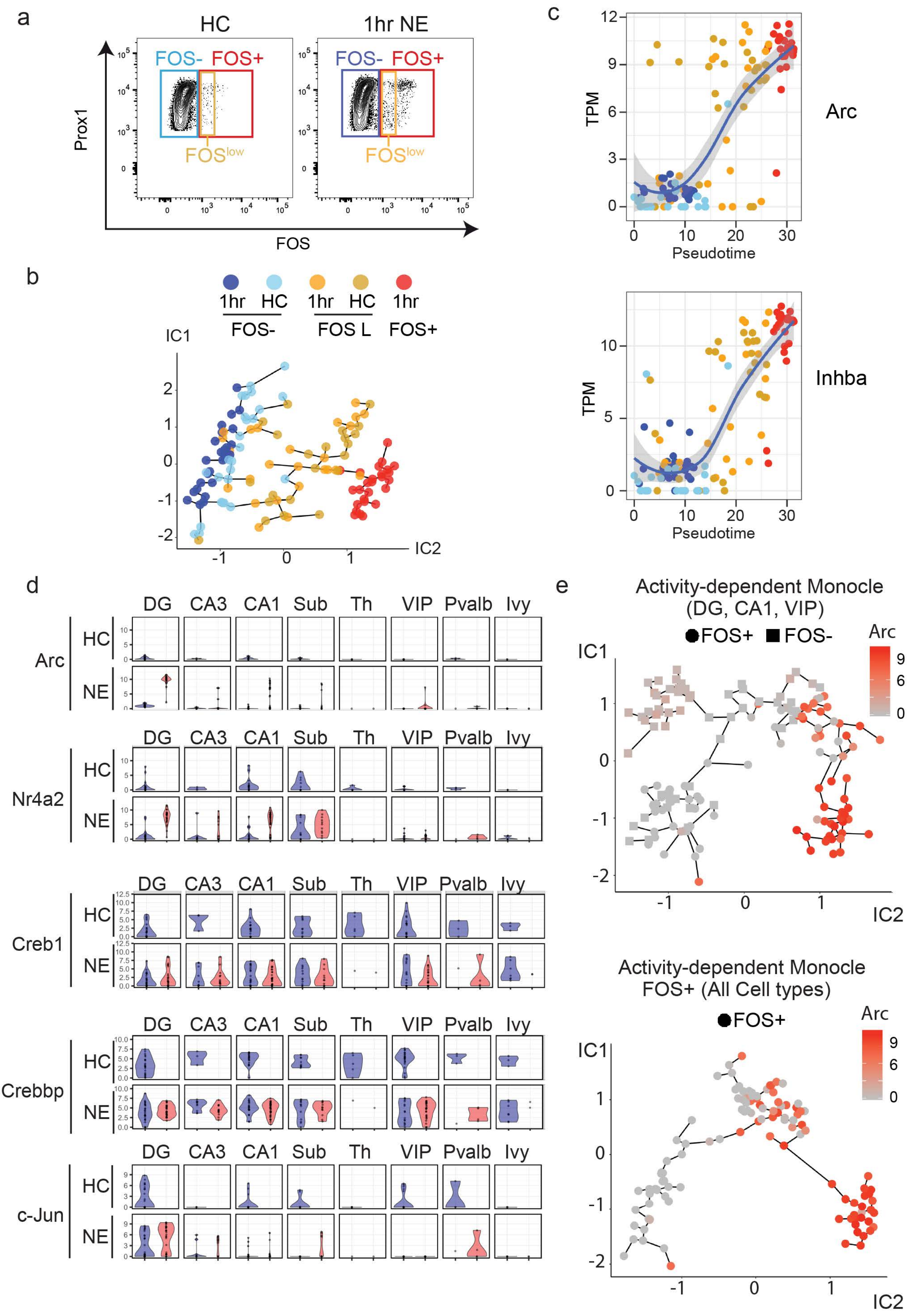

Supplementary Figure 3: ***Activity-induced gene expression***

a) Gating strategy for the FOS low population of DG (PROX1+CTIP2+) neurons based on the FOS+ gates defined in Fig. 1a. b) Monocle ICA plot for FOS-, FOS low, and FOS+ DG neurons from the HC and 1 hr following exposure to a NE. Each nucleus is colored by the respective FOS protein level. IC = Independent Component. c) Expression for two key activity-induced genes, *Arc* and *Inhba*. Each nucleus is colored by the FOS protein level as in b. d) Violin plots for *Arc*, *Nr4a2*, *Creb1*, *Crebbp*, and *c-Jun*for each identified cell type: dentate gyrus (DG), CA3, CA1, subiculum (Sub), thalamus (Th), vasoactive intestinal peptide (VIP), parvalbumin (Pvalb), and ivy neurons; FOS+ (red) and FOS- (blue); and for animals exposed to a NE for 15 min or retained in the HC. Y-axis is log2(TPM+1). e) Monocle ICA plot of DG, CA1, and VIP FOS+ and FOS- neurons (top) or all FOS+ neurons (bottom) colored by the level of ARC expression in TPM from gray to red.

Supplementary Figure 4

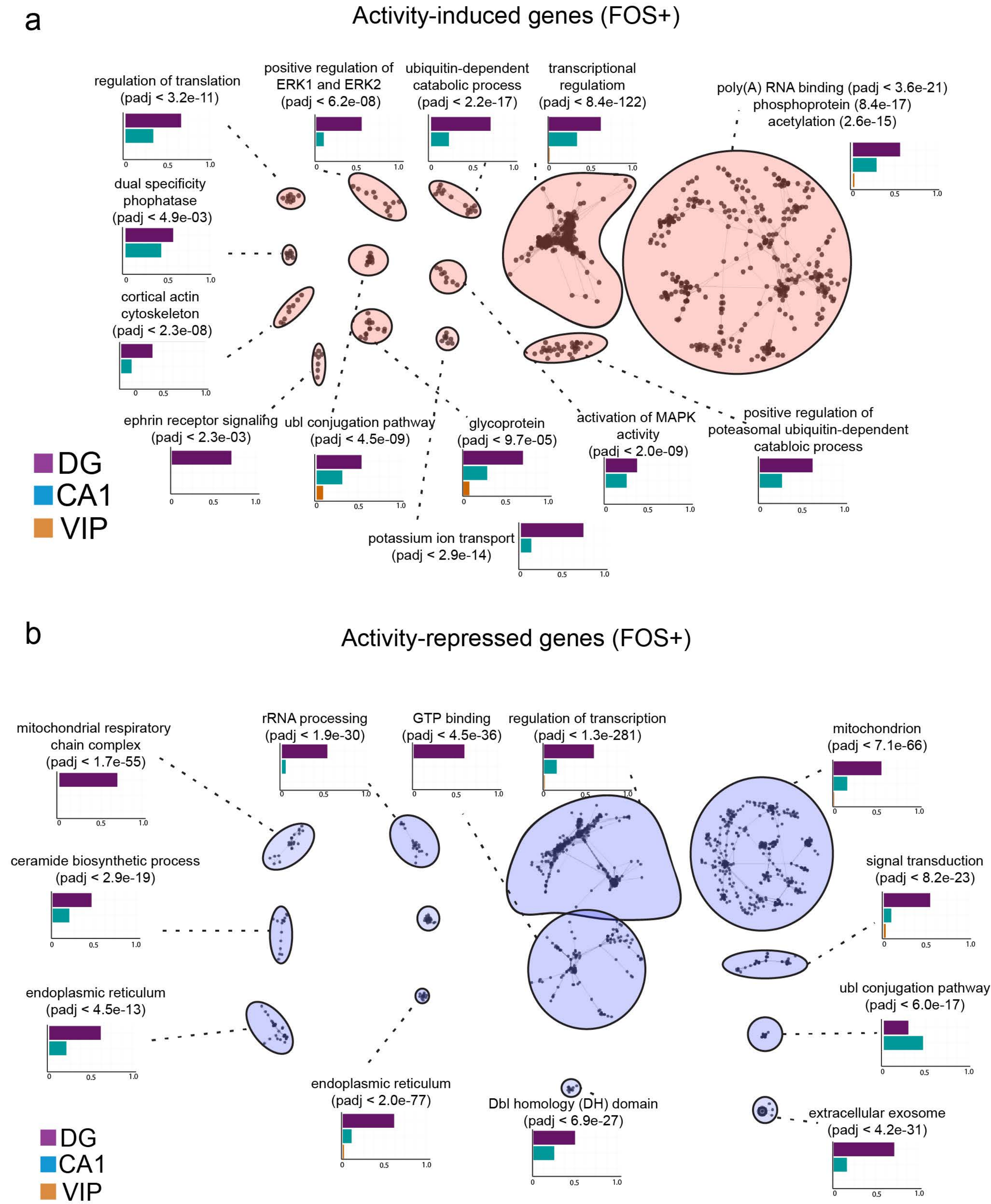

Supplementary Figure 4: ***Functional enrichment of activity-induced gene expression***

a-b) GO terms enriched for all genes that are differentially expressed between FOS+ and FOS- nuclei for each cell type either increasing in FOS+ nuclei (a) or decreasing in FOS+ nuclei (b). Nodes (black circles) are genes and connections (black lines) are the distances between genes as a function of GO annotation. The top enriched term in the cluster is noted next to each cluster. To visualize the proportion of genes within each cluster that are differentially expressed within each cell type, the proportion of genes with a raw Chi-squared p-value < 0.05 was calculated for each cell type and is represented in the nearest bar plot (DG = purple, CA1 = blue, VIP = orange).

Supplementary Figure 5

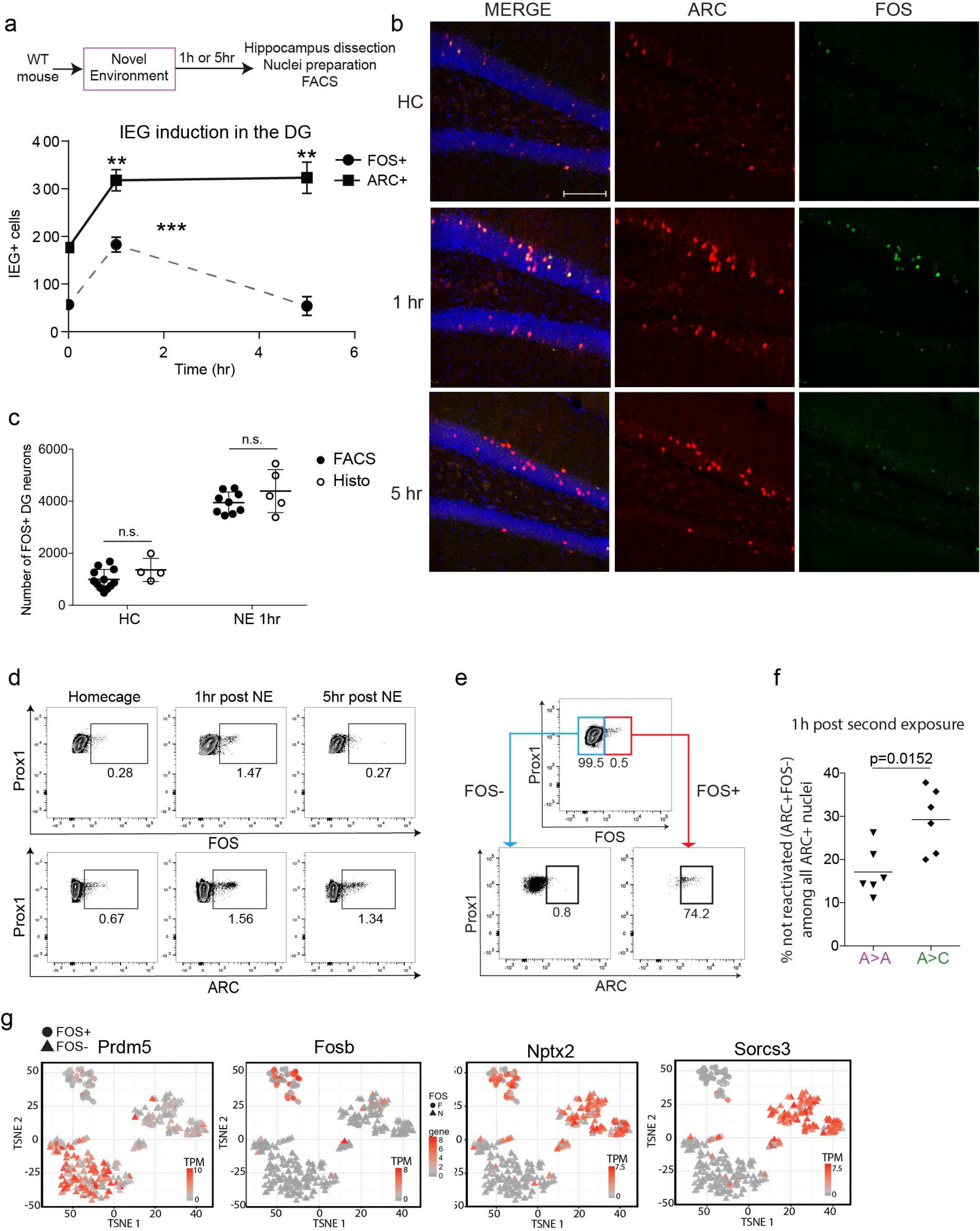

Supplementary Figure 5: ***ARC and FOS in late and reactivated cells***

a) Mice were exposed to a NE for 15 min and perfused 1 or 5 hr later and compared to HC controls. IEG+ cells in the dentate gyrus (DG) were quantified at each time point. Values displayed are mean +/- S.E.M of n=4-5 mice per group. ** p<0.01 vs HC; *** p<0.001 vs HC, one-way ANOVA with Dunnett's multiple comparisons test. b) Representative confocal images of ARC and FOS staining in each group. c) Number of FOS+ DG neurons as detected by FACS and histology, +/- S.D, n=4-12 mice per group, t-test, n.s. = not significant. d) Representative FACS plots for FOS (top) or ARC (bottom) from HC, 1, or 5 hr post NE. f) Percentage of cells that were ARC+FOS- (not reactivated) 1 hr following the second exposure, t-test, n=6. g) T-SNE colored by TPM for representative marker genes for baseline, early, and late gene signatures.

Supplementary Figure 6

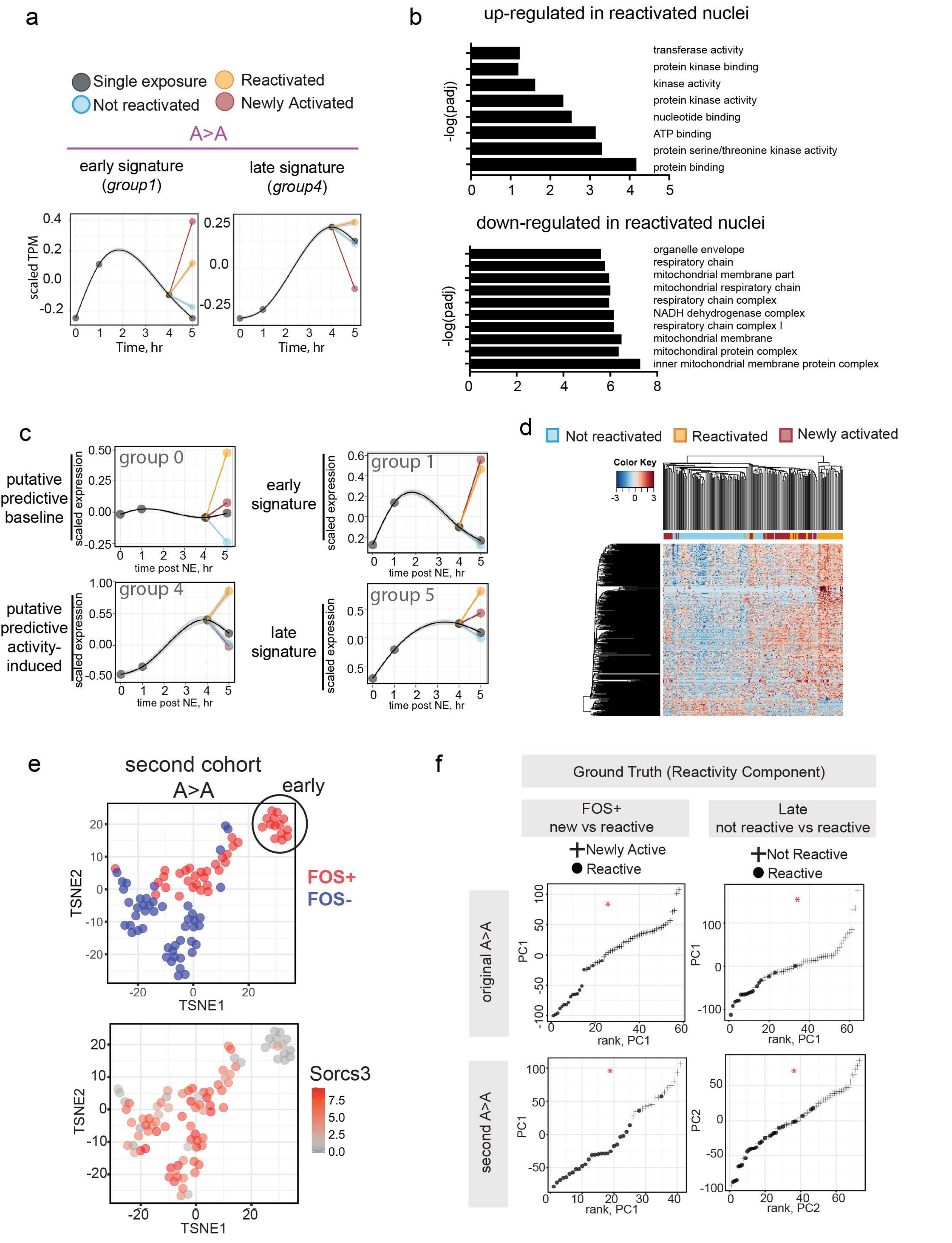

Supplementary Figure 6: ***Gene signatures following the second exposure***

a) Average expression of the early (left) and late (right) gene signatures following re-exposure. The black line marks expression in mice that were exposed to only one context. b) Enrichment of functional terms within genes that are up-regulated (top) and down-regulated (bottom) in reactivated nuclei. c) Average expression profile for putative predictive baseline (top left), putative predictive activity-induced genes (bottom left), early signature genes (top right), and late signature genes (bottom right) for DG nuclei from mice exposed to one context (black circles) or re-exposed to A (red, yellow, and blue circles). d) Heatmap of genes differentially expressed between reactivated and not reactivated nuclei. e) Clustering of nuclei from the second cohort of A>A mice. (Top) t-SNE of all nuclei, a subset of FOS+ nuclei cluster separately (early). (Bottom) Expression of the late expressing gene, *Sorcs3* in TPM. f) Reactivity component for the original (top) and second (bottom) A>A cohorts. Note the separation of reactivated nuclei from either the newly active (left) or not reactive (right) nuclei for each component (red * = PC ~ class p-value < 0.05).

Supplementary table 1: Key resources

REAGENT or RESOURCE	SOURCE	IDENTIFIER
**Antibodies**		
Rabbit polyclonal anti-VIP	Immunostar	#20077 RRID:AB_572270
mouse monoclonal anti-PROX1	EMD Millipore	#MAB5654 RRID:AB_2170714
rat monoclonal anti-CTIP2	Abcam	#ab18465 RRID:AB_2064130
guinea pig polyclonal anti-ARC	Synaptic Systems	#156005 RRID:AB_2151848
goat polyclonal anti-c-FOS	Santa Cruz	sc-52-G RRID:AB_2629503
rabbit polyclonal anti-PROX1	Abcam	#ab101851 RRID:AB_1071221
mouse monoclonal anti-NEUN conjugated to AF488	EMD Millipore	#MAB377X RRID:AB_2149209
donkey anti-rabbit Cy3	Jackson ImmunoResearch	#711-165-152 RRID:AB_2307443
donkey anti-mouse AF488	Jackson ImmunoResearch	#715-545-151 RRID:AB_2341099
donkey anti-rat AF647	Jackson ImmunoResearch	#712-605-153 RRID:AB_2340694
donkey anti-guinea pig Cy3	Jackson ImmunoResearch	#706-165-148 RRID:AB_2340460

donkey anti-goat AF488	Jackson ImmunoResearch	#705-545-147<br>RRID:AB_2336933
donkey anti-mouse-PE	Jackson ImmunoResearch	#715-165-152<br>RRID:AB_2307443
donkey anti-rat-Dylight 405	Jackson ImmunoResearch	#712-475-153<br>RRID:AB_2340681
donkey anti-goat-AF647	Jackson ImmunoResearch	#705-605-147<br>RRID:AB_2340437
**Chemicals, Peptides, and Recombinant Proteins**		
ProtoScript® II Reverse Transcriptase	New England Biolabs	M0368X
Betaine 5M	Sigma	B0300-5VL
RNase Inhibitor (Cloned) 40 U/ L	Ambion	AM2684
2x KAPA HiFi HotStart ReadyMix	KAPA	KK2602
**Critical Commercial Assays**		
Nextera XT DNA Library Preparation Kit	Illumina	FC-121-1031
Picogreen Quant-iT dsDNA assay kit	Invitrogen	P11946
RNAscope® Multiplex Fluorescent Reagent Kit v2	ACD Bio	#323100
RNAscope® Probe – Mm-Sorcs3	ACD Bio	#473421
RNAscope® Probe – Mm-Blnk-C2	ACD Bio	#300031-C2
**Deposited Data**		
Single-nucleus RNA sequencing	Gene expression omnibus	GSE98679

Experimental Models: Organisms/Strains		
C57BL/6 mice	Envigo	C57BL/6NHsd
C57BL/6 mice	Taconic	C57BL/6NTac
**Oligonucleotides**		
OligodT primers:<br>AA GCA GTG GTA TCA ACG CAG AGT ACT TTT TTT TTT TTT TTT TTT TTT TTT TTT TTV N	Integrated DNA technologies	
TSO primers:<br>AAGCAGTGGTATCAACGCAGAGTAC ATrGrG+G	Exiqon	
ISPCR primers:<br>AA GCA GTG GTA TCA ACG CAG AGT	Integrated DNA technologies	
ERCC ExFold RNA Spike-In Mixes	Thermo Fisher Scientific	Cat # 4456739
**Software and Algorithms**		
RSEM	[1]	
Solexa-QA++	[2]	
ROTS	[3]	
randomForest	[4]	
DAVID Bioinformatics	[5]	
GONetwork	https://github.com/saralinker/GONetwork	
Cytoscape	[6]	
Monocle	[7]	
Homer	[8]	

STAMP	[9]	
ROCR	[10]	

1. Li B, Dewey CN. RSEM: accurate transcript quantification from RNA-Seq data with or without a reference genome. *BMC Bioinformatics* **12**, 323 (2011).

2. Cox MP, Peterson DA, Biggs PJ. SolexaQA: At-a-glance quality assessment of Illumina second-generation sequencing data. *BMC Bioinformatics* **11**, 485 (2010).

3. Suomi T, Seyednasrollah F, Jaakkola MK, Faux T, Elo LL. ROTS: An R package for reproducibility-optimized statistical testing. *PLoS Comput Biol* **13**, e1005562 (2017).

4. Liaw AWM. Classification and Regression by randomForest. *R News* **2**, 18-22 (2002).

5. Huang da W, Sherman BT, Lempicki RA. Systematic and integrative analysis of large gene lists using DAVID bioinformatics resources. *Nature protocols* **4**, 44-57 (2009).

6. Lopes CT, Franz M, Kazi F, Donaldson SL, Morris Q, Bader GD. Cytoscape Web: an interactive web-based network browser. *Bioinformatics* **26**, 2347-2348 (2010).

7. Qiu X*, et al.* Reversed graph embedding resolves complex single-cell trajectories. *Nature methods*, (2017).

8. Heinz S*, et al.* Simple combinations of lineage-determining transcription factors prime cis-regulatory elements required for macrophage and B cell identities. *Mol Cell* **38**, 576-589 (2010).

9. Parks DH, Tyson GW, Hugenholtz P, Beiko RG. STAMP: statistical analysis of taxonomic and functional profiles. *Bioinformatics* **30**, 3123-3124 (2014).

10. Sing T, Sander O, Beerenwinkel N, Lengauer T. ROCR: visualizing classifier performance in R. *Bioinformatics* **21**, 3940-3941 (2005).
