# Supplementary material for: A novel environment-evoked transcriptional signature predicts reactivity in single dentate granule neurons
Source: Nat Commun. 2018 Aug 6;9:3084. doi: 10.1038/s41467-018-05418-8 (PMC6079101; doi:10.1038/s41467-018-05418-8)
Supplement: Supplementary file 3 — Description of Additional Supplementary Files [file 41467_2018_5418_MOESM3_ESM.pdf]

Description of Additional Supplementary Files

Supplementary Data 1: ***Cell type-associated genes***
Description: Results of a random forest model examining the association of hippocampal cell type with transcription (TPM ~ cell type). For each cell type we report the estimate, standard error (StdError), t-value, and p-value. Tests identifying genes that are significantly associated with a given cell type below a Bonferroni-corrected alpha = 3.06e-05 are highlighted in green. The importance value for a given gene in aiding cell type discrimination is noted in the RF Importance column.

Supplementary Data 2: ***Cell type classification***
Description: Final assessment of counts of nuclei per cell type based on a combination of hierarchical clustering and random forest classification. N = Total number of cells; N, NE FOS+ = Number of FOS+ nuclei from animals exposed to a novel environment (NE) for 15 min and returned to the home cage (HC) for 1hr; N, NE FOS- = Number of FOS- nuclei from animals exposed to NE for 15 min followed by 1hr in the HC; N, HC FOS- = Number of FOS- nuclei from HC animals. Cluster precision = 1 – out of bag error resulting from random forest analysis using cell-type specific genes (TPM ~ cell type). Pvalb = Parvalbumin expressing interneurons, Th = Thalamus, Sub = Subiculum, DG = Dentate granule neurons.

Supplementary Data 3: ***ROTS differential expression results***
Description: Results of paired differential expression tests using ROTS. All tests with a raw p < 0.05 reported. DG = Dentate granule neuron, NE = novel environment, HC = home cage. The specifics of each test are included on the corresponding worksheet.

Supplementary Data 4: ***Dynamics of activity-induced expression in DG neurons***
Description: All genes were assessed for dynamic patterns of expression following a 15-min exposure to a novel environment (NE). Nuclei were collected from the DG 1 hr, 4 hr, and 5hr following the activating exposure. longterm_groups = Dynamic categories for genes induced in response to a single 15 min exposure to a NE. All values compared to transcription in the HC condition. Group = sample groups with detectable elevated expression compared to HC. All DAVID tabs contain functional enrichment results for the corresponding gene set.

Supplementary Data 5: ***Sample counts***
Description: Nuclei counts (top) and biological replicate counts (bottom) for the samples used in this study. HC = home cage.
